# Marine microalgae *Schizochytrium* demonstrates strong production of essential fatty acids in various cultivation conditions, advancing dietary self-sufficiency

**DOI:** 10.3389/fnut.2024.1290701

**Published:** 2024-05-24

**Authors:** Petra Literáková, Tomáš Zavřel, Diana Búzová, Petr Kaštánek, Jan Červený

**Affiliations:** ^1^Department of Adaptive Biotechnologies, Global Change Research Institute, The Czech Academy of Sciences, Brno, Czechia; ^2^Department of Biochemistry, Faculty of Science, Masaryk University, Brno, Czechia; ^3^EcoFuel Laboratories s.r.o., Prague, Czechia

**Keywords:** docosahexaenoic acid, unicellular eukaryote, growth, optimization, health supplements, bioreactors, process automation

## Abstract

**Introduction:**

Polyunsaturated fatty acids (PUFAs) are essential nutrients that humans obtain from their diet, primarily through fish oil consumption. However, fish oil production is no longer sustainable. An alternative approach is to produce PUFAs through marine microalgae. Despite the potential of algae strains to accumulate high concentrations of PUFAs, including essential fatty acids (EFAs), many aspects of PUFA production by microalgae remain unexplored and their current production outputs are frequently suboptimal.

**Methods:**

In this study, we optimized biomass and selected ω-3 PUFAs production in two strains of algae, *Schizochytrium marinum* AN-4 and *Schizochytrium limacinum* CO3H. We examined a broad range of cultivation conditions, including pH, temperature, stirring intensity, nutrient concentrations, and their combinations.

**Results:**

We found that both strains grew well at low pH levels (4.5), which could reduce bacterial contamination and facilitate the use of industrial waste products as substrate supplements. Intensive stirring was necessary for rapid biomass accumulation but caused cell disruption during lipid accumulation. Docosahexaenoic acid (DHA) yield was independent of cultivation temperature within a range of 28–34°C. We also achieved high cell densities (up to 9 g/L) and stable DHA production (average around 0.1 g/L/d) under diverse conditions and nutrient concentrations, with minimal nutrients required for stable production including standard sea salt, glucose or glycerol, and yeast extract.

**Discussion:**

Our findings demonstrate the potential of *Schizochytrium* strains to boost industrial-scale PUFA production and make it more economically viable. Additionally, these results may pave the way for smaller-scale production of essential fatty acids in a domestic setting. The development of a new minimal culturing medium with reduced ionic strength and antibacterial pH could further enhance the feasibility of this approach.

## 1 Introduction

Polyunsaturated fatty acids (PUFAs) are beneficial for human health and physiology. PUFAs constitute a large group of fatty acids (FAs) that contain more than one double bond (C=C), starting either three (ω-3), six (ω-6), or nine (ω-9) atoms away from the terminal methyl group in their chemical structure. Among the ω-3 PUFAs, α-linoleic acid (18:3n-3; ALA), eicosapentaenoic acid (20:5n-3; EPA), and docosahexaenoic acid (22:6n-3; DHA) are of particular interest, as they have been demonstrated to prevent heart attacks and reduce the risk of cardiovascular disease (1) and inflammation-related events (2).

Due to the limited ability of the human body to synthesize certain FAs, such as EPA or DHA, the primary source of these essential FAs is a well-balanced diet. At present, most ω-3 FAs are produced by the fish industry, particularly through the extraction of fish oil from salmon, mackerel, and herring. However, the current scale of fishing, which has reached a historical maximum and continues to grow, is no longer sustainable (3). Moreover, global warming, which is responsible for the unprecedented increase in ocean temperatures, along with increasing pollution has adverse effects on the growth of many planktonic organisms, which, in turn, negatively affect natural fish populations and ultimately interfere with fish PUFA production. Water contamination by pollutants, such as heavy metals, has already put considerable constraints on the production of fish oil (4).

To protect marine ecosystems and follow the principles of sustainability and climate responsibility, it is necessary to seek alternative PUFA sources. While seeds of plants, such as flax, primrose, and hemp, contain considerable amount of PUFAs (3), these are only short-chain ω-3 PUFAs with little EPA and DHA. Because fish obtain most PUFAs from phytoplankton (5), the production of PUFAs by microalgae has been studied for decades. Many algae strains can produce DHA and EPA (6), and some eukaryotic microalgae, such as *Botryococcus braunii* or *Nannochloropsis* sp., can accumulate up to 70% of lipids in their biomass (7). Marine microalgae, such as *Nannochloropsis* sp. or *Nitzschia* sp., combine the advantage of high DHA accumulation capacity (8) with the possibility of cultivation in salt water, which has the potential to substantially reduce production costs (9) while avoiding the need to compete for scarce freshwater resources.

Of all marine microalgae, thraustochytrids, strains from the order Thraustochytrida are considered to be the most suitable candidates for PUFA production (6). During heterotrophic cultivation, Thraustochytrida can accumulate PUFAs at rates as high as 10 g L^−1^ d^−1^ (10), which is significantly higher compared with other microalgae such as *Nannochloropsis* (11) or *Tisochrysis* (12) during autotrophic or mixotrophic cultivation, as shown in Supplementary Table S1. Interestingly, the production of extracellular enzymes by thraustochytrids has been reported (13, 14), as well as other high-value products such as squalene and carotenoids, with health beneficial properties (15, 16). Squalene, for example, plays a key role in plants and animals as a precursor of many steroids, hormones, and vitamin D synthesis. There is also evidence for the capability of some *Schizochytrium* sp. strains to produce exopolysaccharide, which exhibits a broad spectrum of antiviral activities (17). *Schizochytrium* sp. can produce DHA at the rate of 7–10 g L^−1^ d^−1^ (10, 18). Similarly, the strain *Aurantiochytrium* sp. can produce DHA up to 8.1 g L^−1^ d^−1^ (19). However, such an exceptional PUFA production requires high concentrations of nutrients in the cultivation medium so as to reach cultures with high cell density. A typical cultivation medium for thraustochytrids contains high concentrations of C source (glucose or glycerol), N source (yeast extract, peptone, and sodium glutamate), and micronutrients. Such substrates, however, may increase the price of the final product by as much as 30% (20). There are, however, alternative substrates that have been shown to provide sufficient nutrition. These include spent osmotic solution from the candied fruit industry (21), spent brewery yeast (22), orange peel extract (23), sugar cane molasses, cheese whey, and expired orange juice (24). These alternative extracts can reduce the price of the final oil containing 35–40% of DHA to USD 30 per liter (20). However, an often cited bottleneck in expanding PUFA production by thraustochytrids is the necessity to optimize the cultivation conditions, including nutrient content for every single new isolate (1, 25).

This study focuses on the optimization of biomass and DHA production in one representative *Schizochytrium marinum* AN-4 and one novel *Schizochytrium limacinum* CO3H strain of the order Thraustochytrida (hereafter referred to as AN-4 and CO3H, respectively). It is demonstrated that both of these strains reach stable DHA production under a variety of cultivation conditions, making them ideal candidates for extensive PUFA production. Both strains can grow at pH as low as 4.5. Such a pH level creates a hostile environment for bacteria, thus reducing the risk of bacterial contamination and finally resulting in a more affordable bioproduct. Additional factors that cut the operational costs of DHA production still further included moderate temperature (28°C), only 20% temporal requirements for intensive stirring, and reduced concentrations of nutrients (carbon, nitrogen, and salt) in the cultivation medium. High DHA yields by CO3H, equivalent to those that have been reported for AN-4 (26), are recorded here for the first time. This study demonstrates that both AN-4 and CO3H strains may contribute toward an economically feasible DHA production. It further identifies the factors that are likely to lower operation costs and reduce contamination risks during DHA production by marine microalgae. The presented setup combines automated optimization of growth conditions in a turbidostatic culturing regime with DHA production optimization in batch cultures, amounting to an efficient, fast, and reliable PUFA optimization method.

## 2 Materials and methods

### 2.1 Algal strains

The strains *Schizochytrium marinum* AN-4 and *Schizochytrium limacinum* CO3H were obtained from the culture collection at the Department of Biochemistry and Microbiology at the University of Chemistry and Technology in Prague (Czechia). The stock cultures were maintained in 250 mL Erlenmeyer flasks containing Chang medium (18) supplemented with micronutrients (see Supplementary Table S2 for details), glycerol (50 g L^−1^), and yeast extract (3.6 g L^−1^). The flasks were kept in an Orbital Shaker-Incubator ES-20 (Biosan, Latvia) and were shaken at 150 rpm at 28°C. The stock cultures were replenished weekly with fresh medium.

### 2.2 Experimental setup

#### 2.2.1 Optimization of *Schizochytrium* growth in turbidostat cultivations

**Figure 1:**
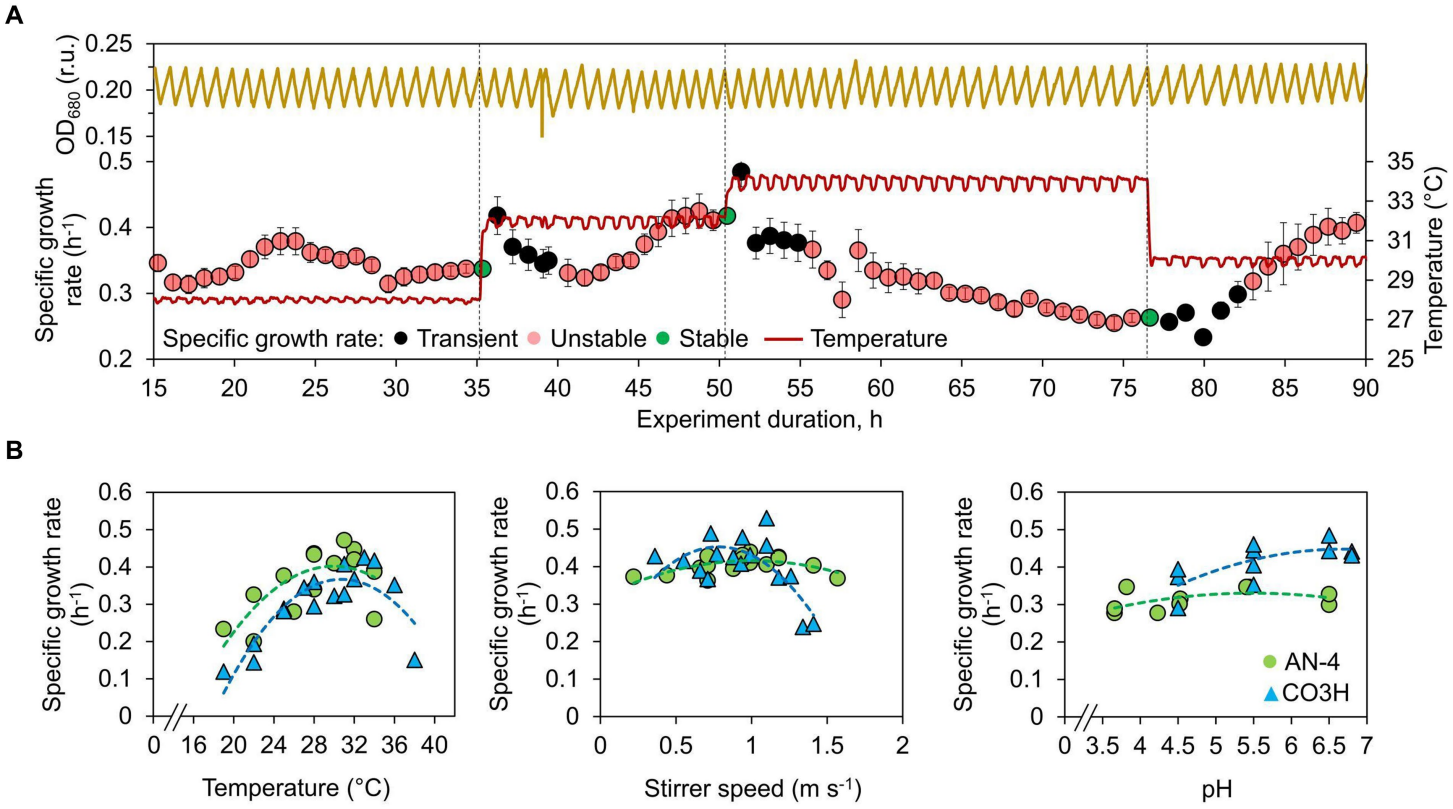
Optimization of *Schizochytrium* growth in a turbidostat. **(A)** Example of temperature optimization. Culture density was kept within a defined OD_680_ range, and a specific growth rate was calculated from OD_680_ increase between the dilution events preceding and following each growth period. After growth stabilization, the conditions were shifted to the next defined level. At the onset of the cultivation condition shift, the growth rate from the initial five periods was considered transient, and growth stability was evaluated starting with the 6^th^ period as described in Equation (1). Growth was evaluated as stable when a linear trend of five subsequent growth rates was <1.5% of the average growth rate, and at the same time, the coefficient of variation was <3%. **(B)** Temperature, stirring speed, and pH dependency of *Schizochytrium* AN-4 (gray circles) and CO3H (black triangles) growth. The values represent all measured replicates, and the dashed lines represent data interpolation by a 2^nd^ order polynomial regression model. A full list of conditions is shown in Supplementary Table S3.

Optimal values of temperature, pH, and shear stress for biomass accumulation by both *Schizochytrium* strains were identified in FMT-150 photobioreactors [400 mL, PSI, Czechia (27, 28)] in a turbidostat regime. Culture density was maintained within a defined density range by diluting the culture suspension with fresh medium in response to the continuously monitored optical density of the culture suspension at 680 nm (OD_680_). The OD_680_ range was set to 0.18–0.21, corresponding to 2 × 10^9^ cells L^−1^. The initial OD_680_ was ~0.05 (5 × 10^8^ cells L^−1^). Specific growth rates were evaluated online by the increase in the OD_680_ signal at each dilution step using an exponential regression model (29). This allowed the stability of *Schizochytrium* growth to be evaluated virtually in real time: a typical time window necessary for the application of the regression model is between 30 and 45 min. After a shift in cultivation conditions, the initial five dilution steps were considered transient, and the specific growth rate was evaluated starting with the 6th dilution step (Figure 1A) using an own-developed algorithm implemented into FMT-150 operating software. The growth was considered stable (i.e., fully acclimated to a given set of conditions, Figure 1B) when the following criteria were met:

a) The linear trend stability of five subsequent growth rates (μ), as calculated from OD_680_ increase within turbidostat dilutions was <1.5% of the average growth rate calculated as follows:


(1) $$trendStability=\frac{m}{\frac{\sum_{i=1}^{5}\mu_{i}}{5}}\cdot 100$$

where m is the linear trend slope calculated using the standard linear regression method and.

b) coefficient of variation (CV) was <3%.

In the turbidostat regime, the *Schizochytrium* cultures (300 mL) were bubbled by air (400 mL min^−1^) and cultivated under the temperature of 19–38°C and a pH of 3.7–6.8 and agitated by rotations of a magnetic stirrer bar with circular velocities of 0.22–1.57 m s^−1^. pH was maintained at desired levels by the addition of 0.1 N NaOH and HCl via peristaltic pumps (PSI, Czechia). The pH optimization was therefore carried out in a combined turbidostat and pH-stat regime. A summary of all cultivation conditions used during the turbidostat experiments is shown in Supplementary Table S3.

#### 2.2.2 Optimization of PUFA production in batch cultivations

After the initial optimization of *Schizochytrium* growth in a turbidostat, the biomass and FA production were quantified in a batch regime using identical bioreactors (400 mL). The conditions for the batch cultivation were based on the turbidostat experiments: the temperature range of 28–34°C, pH of 4.5–6.5, stirrer bar circular velocities of 0–1.1 m s^−1^, Biosal^®^ (Zangrando, Italy) or Chang (18) medium both with and without microelement supplementation (as shown in Supplementary Table S2), with glucose or glycerol in the concentrations of 15, 25, or 50 g L^−1^, and yeast extract in the concentrations of 3.6 or 7.2 g L^−1^ constituting C and N sources, respectively. A detailed list of cultivation conditions is shown in Supplementary Table S4. All cultures (400 mL) were bubbled by air (400 mL min^−1^). The effect of oxygen increase on DHA production was tested by aeration with 50% O_2_ in the air mixture (medical quality, Siad Czechia) over the initial 24–96 h. The cells were cultivated in the batch regime for 190 h. Samplings for biomass and FA content were performed between 48 and 190 h.

### 2.3 FA composition and productivity

Lipids in the microalgae biomass were quantified following the procedure proposed by Liang et al. (30). In brief, the dried cell pellet was flooded with methanol and disrupted with beads using a bead-beater. The mixture was transferred to a 50 mL glass centrifuge tube, mixed with chloroform (chloroform:methanol ratio 2:1, v/v), vortexed, and allowed to stand for 24 h. Thereafter, the tube was centrifuged, the supernatant was collected, the solvent was vaporized, and the oil remaining in the flask was weighed.

The FAs in lipid extracts were converted to fatty acid methyl esters (FAMEs) by hydrolysis and methylation in a 0.5 mL of 0.5 M methanolic sodium hydroxide solution for 30 min at 80°C. Subsequently, after the mixture had been allowed to cool down to laboratory temperature, 200 μL of 14% BF3 (boron trifluoride) in methanol (Sigma–Aldrich, United States) was added directly to the mixture of fatty acids and 0.5 M methanolic sodium hydroxide in the reaction vials, and the vials were re-heated and kept at 80°C for additional 30 min. The resulting FAMEs were transferred to heptane and analyzed using an Agilent 6890 Plus series II gas chromatograph (Agilent Technologies, United States) coupled with a flame ionization detector (GC/FID) on an SP-2560 column (100 m × 0.25 mm I.D., 0.20 μm). Individual FAMEs were quantified using external commercial standards GLC-10 and GLC-30 (Supelco 37 Component FAME Mis, Supelco, United States). The GC/FID conditions were as follows: oven 140°C (5 min), 4°C min^−1^ to 240°C (15 min); injector 250°C; detector 260°C; helium carrier gas, 20 cm s^−1^ at 175°C; injection 1 μL with 100:1 split.

Biomass production by *Schizochytrium* strains was determined by the assessment of cellular dry weight using analytical balances (Sartorius, Germany).

### 2.4 Statistics

The effect of the cultivation conditions on DHA productivity was evaluated through a factorial ANOVA followed by Tukey’s HSD *post-hoc* test. Variables failing the ANOVA normality or homogeneity of variance assumptions (using Lilliefors, Shapiro–Wilk, Bartlett’s, Cochran’s, and Hartley’s tests, respectively) were transformed before the ANOVA testing, using log, square root, and arcsine transformations. Statistical testing was performed in Statistica software (Tibco, United States).

## 3 Results

### 3.1 Optimization of *Schizochytrium* growth in a turbidostat

Throughout all turbidostat experiments, the specific growth rates of both *Schizochytrium* strains varied between 0.12 and 0.53 h^−1^ (doubling time 5.7–1.3 h). The initial temperature optimization revealed a temperature optimum of 28–34°C, saturating *Schizochytrium* growth by more than 95% (Figure 1B).

Additional evaluation concerned the effect of stirring and pH. Increasing the stirring rate improved the growth of both strains (0.4 h^−1^), but only up to a point, beyond which the strains were exposed to shear stress. The CO3H strain was more sensitive to shear stress than AN-4: the optimum stirrer bar circular velocity was identified as 0.7–1 m s^−1^ for both strains. The stirring rate of 1.4 m s^−1^ reduced CO3H growth by 45%, whereas the corresponding decrease for AN-4 was only 3% (Figure 1B).

The decrease of pH from 6.5 to 4.5 reduced the growth rate of the AN-4 strain by 2%, and the growth rate of CO3H reduced by 15% (Figure 1B). Despite this slight growth inhibition, pH 4.5 was set for batch cultivations intended for the evaluation of FA production. Low pH is known to reduce bacterial contamination [for a review, see (31)], which was further confirmed in our experiments by the fact that no bacterial contamination occurred over the course of the batch experiments with pH set to 4.5 (lasting up to 190 h).

### 3.2 DHA productivity by *Schizochytrium* batch cultures

**Figure 2:**
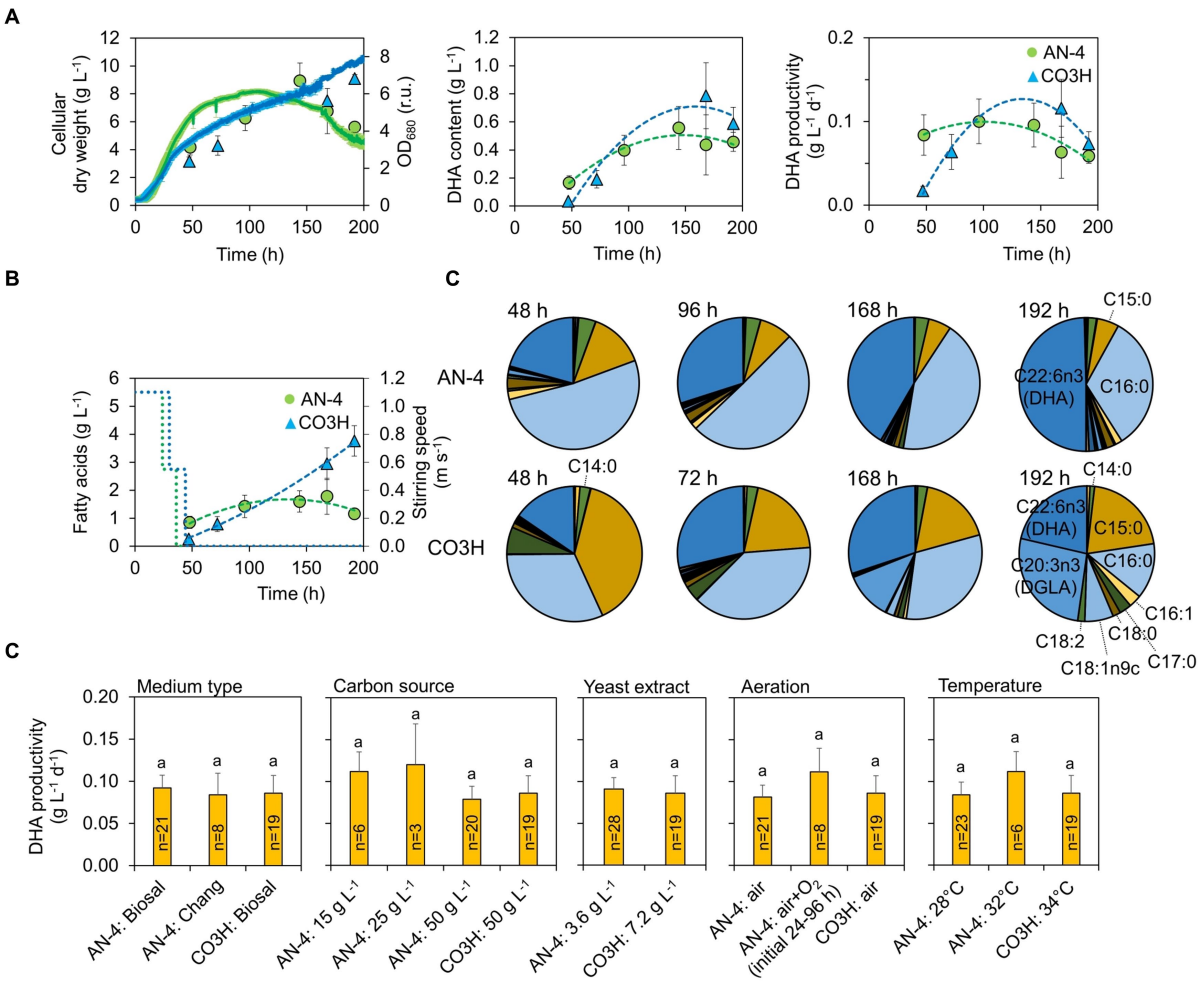
Productivity of biomass, FAs, and DHA in AN-4 and CO3H strains during batch cultivation. **(A)** Biomass growth and DHA productivity. **(B)** Stirring intensity during batch cultivations and FA content in both strains. **(C)** FA profiles in both strains. **(D)** Effect of cultivation conditions on DHA productivity. The letters above the columns indicate statistically significant groups (ANOVA followed by Tukey’s HSD *post-hoc* test). All values in **(A–D)** represent averages±SE (*n* = 3–21). For clarity, the values in **(C)** are listed without error bars.

**Figure 3:**
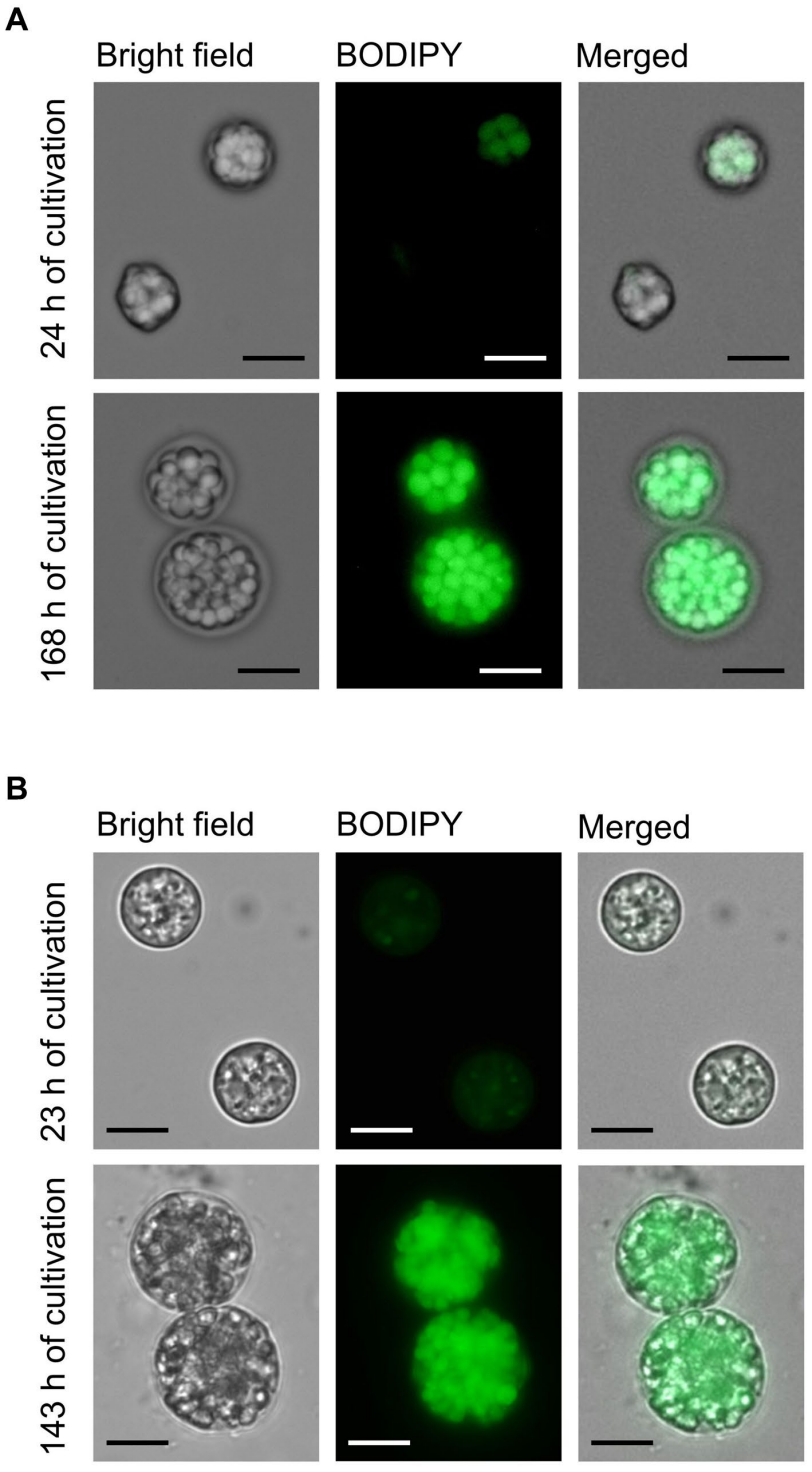
Microscopic images of AN-4 **(A)** and CO3H **(B)** cells in the exponential (23–24 h) and stationary (143–168 h) phases of batch cultivation. After their withdrawal from the bioreactor, the cells were fixed by glutaraldehyde (final concentration 0.25%) and stained by BODIPY to visualize lipid fluorescence. The cells were photographed by a Zeiss Axio Imager M2 fluorescence microscope. BODIPY fluorescence representing lipids was measured with a 480 nm excitation laser and 535/30 nm emission filter sets. The scale bar represents 10 μm.

The productivity of FAs including DHA in both strains was evaluated in a batch regime (Figure 2A). Both strains were cultivated at pH 4.5 with a gradually decreasing speed of the magnetic stirrer bar (Figure 2B). A complete list of conditions set during the batch experiments is shown in Supplementary Table S4. Across all the tested conditions, the maximal biomass productivity for strains AN4 and CO3H was 15 g L^−1^ and 13 g L^−1^, maximal FA content was 520 mg g DW^−1^ and 690 mg g DW^−1^, maximal DHA content was 150 mg g DW^−1^ and 254 mg g DW^−1^, and maximal DHA productivity was 0.24 g L^−1^ d^−1^. And 0.36 g L^−1^ d^−1^, respectively (Figure 2B). The DHA content in the AN-4 strain was increased in the interval between 48 and 192 h, and at the end of the cultivation, DHA accounted for 50% of the entire FA content (Figure 2C). The CO3H strain contained only 15–30% of DHA owing to a high concentration of dihomo-γ-linolenic acid (DGLA, Figure 2C). However, due to faster biomass and total FA accumulation over time, both strains had a comparable DHA productivity (Figure 2A). High FA content in both strains in the linear/stationary growth phase was further confirmed by fluorescence microscopy (Figure 3).

No differences in DHA productivity were detected under varying cultivation parameters, including medium type (Chang et al. (18) vs. Biosal®), C source concentration (15–50 g L^−1^), aeration type (air vs. air+O_2_), N source concentration (yeast extract 3.6 vs. 7.2 g L^−1^), and temperature (28–32–34°C; Figure 2D). Such robust DHA productivity is related to relatively stable productivity of biomass (< 2.2 g L^−1^ d^−1^); the DHA content was varying throughout all tested experimental conditions and growth phases considerably (Supplementary Table S5).

## 4 Discussion

Online evaluation of specific growth rate combined with the feedback-loop control of cultivation condition shift during the turbidostat growth optimization was a significant improvement over previous studies, in which a 24-h acclimation period was typically required to ensure full metabolic acclimation (29, 32, 33). In the present case, the stability of the growth rate (which reflects on metabolic acclimation) was determined after each dilution event with a sub-hour frequency as a result of the fast growth rate of both heterotrophic organisms. This setup allowed to significantly reduce the early detection of acclimation time to periods as short as 6 h.

The optimal temperature range for the growth of both strains was identified at 28–34°C, which was slightly higher compared with other *Schizochytrium* strains (34, 35). This shift is likely related to the cultivation setup: the turbidostat enabled full metabolic acclimation, whereas during batch cultivations, as used in previous studies, the conditions were constantly shifting due to fluctuating availability of nutrients, which only allows the achievement of metabolic pseudo-steady states (28).

The stirring circular velocity of 0.88 m s^−1^ resulted in a shear stress of ≤12 Pa (36). The shear stress of >2 Pa is generally considered to be harmful to algae (37). Indeed, stirring improves gas exchange between the cells and the culture medium and delays oxygen limitation (38), which is a limiting factor for DHA production (18). Here, however, even O_2_ concentration as high as 50% during the initial growth phase (≤96 h) did not improve DHA production (Figure 2D). Therefore, cultivation was performed with ambient air aeration and stirring gradually decreasing to zero, in order to avoid cell damage in the lipid production phase (as shown in Supplementary Figure S1).

*Schizochytrium* tolerance to pH as low as 3.7 is beneficial for the control of bacterial contamination. Most bacterial strains are neutrophils (39) and need to expend substantial amount of energy to survive in an acidic environment (40). During the batch cultivation performed in this study, pH 4.5 was low enough to prevent the *Schizochytrium* cultures from being outgrown by bacteria.

The maximal achieved DHA productivity of 0.36 g L^−1^ d^−1^ for the strain CO3H was much lower compared with the maximal documented DHA production rates of 10 g L^−1^ d^−1^ (10). However, high DHA productivity is typically related to exceptionally high culture densities [as high as 171 g L^−1^ (10)] due to high input of C and N sources during batch or fed-batch cultivations (19). The present study used only up to 50 g L^−1^ of glucose or glycerol (C source) and 7.2 g L^−1^ of yeast extract (N source) in simple batch cultivations, which was significantly less than the concentrations reported in previous studies (productivities achieved by different thraustochytrid strains described in the recent literature are shown in Supplementary Table S1).

However, instead of maximizing DHA production, this study aims to demonstrate that DHA can be produced at rates that are still high enough to fulfill the requirements of the human diet [0.25–0.5 g per person per day (41)] even in pure salt water with the addition of low amount of C and N sources. It has been established that the novel CO3H strain, when cultivated in a small-scale bioreactor, can produce up to 0.36 g L^−1^ d^−1^ of DHA. This amount is sufficient to meet the daily needs of an average adult by cultivation in a 1 L bioreactor under conditions suitable for home-scale production. Given the increasing interest in self-sufficient domestic production, the recent approval of *Schizochytrium* strains as a novel food source containing DHA- and EPA-rich oils [according to EU Novel Food Regulation 2022/1365 (42)], the emergence of affordable culturing systems (43), and the production of microalgae as a dietary supplement at home will likely become more feasible in the near future. This study further confirms this potential by introducing optimized conditions for producing DHA-rich biomass using low antibacterial pH and minimal medium composed of affordable materials readily available in the market, with the final price *ca.* 0.7 EUR L_medium_^−1^, which is 42% of a typical price for the standard Cheng recipe (18), assuming a comparable source and quality of the chemicals employed. However, the main challenge is still posed by ensuring safe processing of the biomass to be suitable for human consumption, in addition to the fact that algae-based food products are still relatively new to consumers and both their understandability and acceptability, as well as the regulatory framework for algae-based products, are still developing. To further encourage consumer acceptance, additional research is necessary to appreciate the lasting effects that the consumption of algal products has on human health and explore methods of addressing sensory drawbacks, such as the taste and odor of marine algae products.

## 5 Conclusion

The optimization of cultivation conditions for one representative *Schizochytrium* strain (AN-4) and one novel *Schizochytrium* strain (CO3H) revealed that stable production of PUFAs, such as DHA, can be achieved under a broad range of conditions and reduced nutrition requirements. While the optimized production has not reached yields recorded for other *Schizochytrium* strains in different culturing systems and more nutrient-rich media, these results attested to the general usability of *Schizochytrium* strains under home-scale cultivation conditions and, for the first time, demonstrate the possibility of cultivating *Schizochytrium* at low, naturally antibacterial pH that could facilitate industrial scale heterotrophic cultivations, in which contamination risk is a major concern.

## Data availability statement

The original contributions presented in the study are included in the article/Supplementary material, further inquiries can be directed to the corresponding author.

## Author contributions

PL: Conceptualization, Investigation, Methodology, Validation, Writing – original draft. TZ: Validation, Writing – original draft, Formal analysis, Visualization. DB: Visualization, Conceptualization, Data curation, Methodology, Writing – review & editing. PK: Conceptualization, Methodology, Writing – review & editing, Funding acquisition, Project administration, Resources, Supervision. JČ: Conceptualization, Funding acquisition, Methodology, Project administration, Resources, Supervision, Writing – review & editing, Data curation, Formal analysis, Investigation, Software, Visualization.
