# Supplementary material for: Marine microalgae Schizochytrium demonstrates strong production of essential fatty acids in various cultivation conditions, advancing dietary self-sufficiency
Source: Front Nutr. 2024 May 24;11:1290701. doi: 10.3389/fnut.2024.1290701 (PMC11157098; doi:10.3389/fnut.2024.1290701)
Supplement: Supplementary file 1 [file Data_Sheet_1.PDF]

Supplementary Material

1 Supplementary Figures and Tables

1.1 Supplementary Tables

This is a provisional file, not the final typeset article

Supplementary Table 1 Productivities of DHA, EPA and biomass of Thraustochytrids and other microalgae strains, as reported in literature, in comparison with the current study. n.a.: not available

Reference	Strain	DHA Productivity [$g L^{-1} d^{-1}$]	DHA Content [$g L^{-1}$]	DHA Content [% DW]	EPA Content [$g L^{-1}$]	EPA Content [% DW]	Biomass Productivity [$g L^{-1} d^{-1}$]	Biomass Content [$g L^{-1}$]	Temperature [°C]	C source [$g L^{-1}$]	N source [$g L^{-1}$]	Cultivation type
Humhal et al. 2019	*Schizochytrium marinum AN4*	1.43	n.a.	48	n.a.	n.a.	4.5	16	23	40	4	heterotrophic
Humhal et al. 2017	*Schizochytrium marinum AN4*	0.86	n.a.	52	n.a.	n.a.	3.3	25	23	50	0-7.2	heterotrophic
Abad et al. 2015	*Aurantiochytrium limacinum SR21 (ATCC MYA-1381)*	1.44	1.3	n.a.	n.a.	n.a.	n.a.	9	n.a.	10	2	heterotrophic
Ethier et al. 2011	*Schizochytrium limacinum SR 21 (ATCC MYA-1381)*	0.52	n.a.	31	n.a.	n.a.	3.5	12	25	15 - 120	5	heterotrophic
Huang et al. 2012	*Aurantiochytrium limacinum SR 21 (ATCC MYA-1381)*	2.94	20.3	73	n.a.	n.a.	n.a.	62	22	20 - 100	20 - 40	heterotrophic
Li et al. 2015	*Aurantiochytrium limacinum SR21 (ATCC)*	8.09	32.4	n.a.	n.a.	n.a.	n.a.	88	25	10 / 40	25	heterotrophic
Ren et al. 2014	*Schizochytrium sp. CCTCC M209059*	3.05	20.0	46	n.a.	n.a.	n.a.	116	28	50	6 - 24	heterotrophic
Wang et al. 2020	*Schizochytrium sp. S31*	0.24	n.a.	n.a.	n.a.	n.a.	1.9	13	25	20	9 – 15	heterotrophic
Fan et al. 2001	*Thraustochytrids sp.*	n.a.	2.8	41	n.a.	n.a.	n.a.	14	25	n.a.	10	heterotrophic
Sahin et al. 2018	*Schizochytrium sp S31 (ATCC 20888)*	n.a.	0.6	40	n.a.	n.a.	n.a.	6	27	n.a.	n.a.	heterotrophic
Perveen et al. 2006	*Thraustochytrid like microorganism 12B*	2.80	6.8	48	n.a.	n.a.	n.a.	31	28	50 - 150	20	heterotrophic
Chang et al. 2013	*Schizochytrium sp S31 ATCC*	7.22	28.9	n.a.	n.a.	n.a.	n.a.	151	28	85	50	heterotrophic
Chi et al. 2009	*Schizochytrium limacinum SR 21 (ATCC MYA-1381)*	n.a.	6.6	19	n.a.	n.a.	n.a.	38	25	100	1	heterotrophic
Qu et al. 2011	*Schizochytrium sp. HX-308 (CCTCC M 209059)*	2.66	17.7	47	n.a.	n.a.	n.a.	93	25	120	0.4	heterotrophic
Ren et al. 2010	*Schizochytrium sp. CCTCC M209059*	2.86	15.8	49	n.a.	n.a.	n.a.	71	25	120	0.4	heterotrophic
Ganuza et al. 2008	*Schizochytrium sp. G13/2S*	3.00	n.a.	n.a.	n.a.	n.a.	31.2	63	27	150	n.a.	heterotrophic
Shafiq et al. 2020	*Schizochytrium limacinum SR21 (ATCC MYA-1381)*	3.84	23.0	n.a.	n.a.	n.a.	n.a.	146	30	10	n.a.	heterotrophic
Hu et al. 2021	*Schizochytrium sp. CCTCC M209059*	n.a.	10.4	48	n.a.	n.a.	n.a.	69	28-35	50	1.5 – 20	heterotrophic
Nazir et al. 2020	*Aurantiochytrium sp. SW1*	2.10	12.7	46	n.a.	n.a.	6.9	42	28	40	2	heterotrophic
Russo et al. 2021	*Aurantiochytrium mangrovei (RCC893)*	0.48	n.a.	n.a.	n.a.	n.a.	3.9	12	20-36	20	4	heterotrophic
Aini et al. 2022	*Aurantiochytrium limacinum SR 21 (ATCC MYA-1381)*	0.96	5.8	22	n.a.	n.a.	4.4	26	25	64	5	heterotrophic
Abdel-Wahab et al. 2022	*Aurantiochytrium sp. YB-05*	1.80	7.2	17	n.a.	n.a.	10.8	43	26	10 – 80	10	heterotrophic
Patel et al. 2020	*Schizochytrium limacinum SR21*	3.38	10.2	35	n.a.	n.a.	9.7	37	25	30 - 120	3 – 12	heterotrophic
Chauhan et al. 2023	*Aurantiochytrium sp.*	0.27	1.3	34	0.04	n.a.	1.5	7	26	10 – 40	n.a.	heterotrophic
Menegol et al. 2019	*Nannochloropsis gaditana B-3*	n.a.	n.a.	n.a.	0.05	4.2	0.3	1	12 – 21	1 – 15	n.a.	mixotrophic
Thurn et al. 2022	*Tisochrysis lutea , Microchloropsis salina*	0.01	0.1	2	0.10	2.1	0.6	5	25	n.a.	n.a.	autotrophic
This study	*Schizochytrium marinum AN4*	0.24	1.1	9	n.a.	n.a.	2.2	15	28-32	15 – 50	3.6	heterotrophic
This study	*Schizochytrium limacinum CO3H*	0.36	2.9	25	n.a.	n.a.	1.2	13	34	50	7.2	heterotrophic

This is a provisional file, not the final typeset article

Supplementary Table 2 Composition of medium used for cultivation of *Schizochytrium* stock cultures.

**Chang medium**	**Final concentration (g L^{-1})**
$(NH_4)_2SO_4$	1
KH_2PO_4	3
Na_2SO_4	12
$MgSO_4$	5
K_2SO_4	7
KCl	2
Micronutrients	
$CaCl_2$	5×10^{-2}
$MnCl_2$	5×10^{-3}
$ZnSO_4$	5×10^{-3}
$CuSO_4$	8×10^{-4}
Na_2MoO_4	1.6×10^{-5}
$NiSO_4$	8×10^{-4}
$FeSO_4$	1×10^{-5}
CoCl2	6.6×10^{-5}
Thiamine	7.6×10^{-4}
Vitamin B12	1.2×10^{-3}
Ca panthotenate	2.56×10^{-2}

This is a provisional file, not the final typeset article

Supplementary Table 3 Cultivation conditions set during turbidostat cultivations of *Schizochytrium* strains AN4 and CO3H.

	AN-4	CO3H
**pH optimization**		
Temperature (°C)	28	34
pH	3.6 - 6.5	4.5 - 6.8
Stirring speed (m s^{-1})	1.1	1.1
Medium	Chang	Biosal
Yiest extract (g L^{-1})	3.6	7.2
Glucose (g L^{-1})	25	-
Glycerol (g L^{-1})	-	50
Microelements + vitamins	No	Yes
**Temperature optimization**		
Temperature (°C)	19 - 34	19 - 38
pH	4.5	4.5
Stirring speed (m s^{-1})	1.1	1.1
Medium	Chang	Biosal
Yiest extract (g L^{-1})	3.6	7.2
Glucose (g L^{-1})	25	-
Glycerol (g L^{-1})	-	50
Microelements + vitamins	No	Yes
**Shear stress optimization**		
Temperature (°C)	28	34
pH	4.5	4.5
Stirring speed (m s^{-1})	0.22 - 1.57	0.36 - 1.34
Medium	Biosal	Biosal
Yiest extract (g L^{-1})	3.6	7.2
Glucose (g L^{-1})	25	-
Glycerol (g L^{-1})	-	50
Microelements + vitamins	No	Yes

This is a provisional file, not the final typeset article

Supplementary Table 4. Summary of the conditions used during batch cultivation.

**Batch cultivations**	**AN-4**	**CO3H**
Temperature (°C)	28 / 32	34
pH	4.5-6.5	4.5
Stirring speed (m s^{-1})	1.1 (24 h) / 0.55 (12 h) / 0 (rest of cultivation)	1.1 (30 h) / 0.55 (12 h) / 0 (rest of cultivation)
Medium	Chang/Biosal	Biosal
Yiest extract (g L^{-1})	3.6	7.2
Glucose (g L^{-1})	25	-
Glycerol (g L^{-1})	15 - 50	50
Microelements + vitamins	Yes / No	Yes
Bubbling gas type	Air / Air+O_2 50%	Air
Sampling time (h)	48-190	48-190

This is a provisional file, not the final typeset article

Supplementary Table 5. List of all fatty acids determined in this work.

Systematic name	Common name	Abbreviation	*Schizochytrium limacinum CO3H* [mg gDW^{-1}]	*Schizochytrium marinum AN4* [mg gDW^{-1}]
Butanoic acid	Butyric acid	C04:0	<0.1 - <0.1	<0.1 - <0.1
Hexanoic acid	Caproic acid	C06:0	<0.1 - 0.8	<0.1 - 1.5
Octanoic acid	Caprylic acid	C08:0	<0.1 - <0.1	<0.1 - <0.1
Decanoic acid	Capric acid	C10:0	<0.1 - <0.1	<0.1 - 4.7
Undecanoic acid	Undecylic acid	C11:0	<0.1 - 0.1	<0.1 - <0.1
Dodecanoic acid	Lauric acid	C12:0	<0.1 - 0.5	<0.1 - 4.2
Tridecanoic acid	Tridecylic acid	C13:0	<0.1 - 5.0	<0.1 - 1.1
Tetradecanoic acid	Myristic acid	C14:0	<0.1 - 16.7	1.3 - 16.8
cis-9-Tetradecenoic acid	Myristoleic acid	C14:1	<0.1 - 0.6	<0.1 - 0.6
Pentadecanoic acid	Pentadecylic acid	C15:0	6.4 - 190.7	1.6 - 77.7
Pentadecenoic acid		C15:1	<0.1 - <0.1	<0.1 - <0.1
Hexadecanoic acid	Palmitic acid	C16:0	2.2 - 341.5	14.8 - 194.1
Hexadecenoic acid	Palmitoleic acid	C16:1	<0.1 - 16.5	<0.1 - 13.2
Heptadecanoic acid	Margaric acid	C17:0	<0.1 - 17.3	<0.1 - 4.0
cis-10-Heptadecanoic acid		C17:1	<0.1 - 0.1	<0.1 - 1.0
Octadecanoic acid	Stearic acid	C18:0	<0.1 - 56.4	0.6 - 15.8
cis-11-Octadecanoic acid	Vaccenic acid	C18:1n11c	<0.1 - 1.1	<0.1 - 5.1
cis-9-Octadecenoic acid	Oleic acid	C18:1n9c	<0.1 - 46.2	<0.1 - 1.0
trans-9-Octadecenoic acid	Elaidic acid	C18:1n9t	<0.1 - 0.1	<0.1 - 0.2
all cis-9,12-Octadecadienoic acid	Linoleic acid	C18:2n6c	<0.1 - 10.1	<0.1 - 0.8
all trans-9,12-Octadecadienoic acid	Linolelaidic acid	C18:2n6t	<0.1 - 0.1	<0.1 - 0.1
all cis-9,12,15-Octadecatrienoic acid	α-Linolenic acid	C18:3n3	<0.1 - 0.5	<0.1 - 0.4
all cis-6,9,12-Octadecatrienoic acid	γ-Linolenic acid	C18:3n6	<0.1 - 0.5	<0.1 - 0.4
Eicosanoic acid	Arachidic acid	C20:0	<0.1 - 0.8	<0.1 - 1.3
cis-11-Eicosenoic acid		C20:1n9	<0.1 - 0.3	<0.1 - 0.1
all cis-11,14-Eicosadienoic acid		C20:2	<0.1 - 1.0	<0.1 - 0.5
all cis-11,14,17-Eicosatrienoic acid		C20:3n3	<0.1 - 300.1	<0.1 - 10.1
all cis-8,11,14-Eicosatrienoic acid	Dihomogammalinolenic acid	C20:3n6	<0.1 - 0.8	<0.1 - 1.2
all cis-5,8,11,14-Eicosatetraenic acid	Arachidonic acid	C20:4n6	<0.1 - 0.9	<0.1 - 1.5
all cis-5,8,11,14,17-Eicosapentenoic acid	EPA	C20:5n3	<0.1 - 2.8	<0.1 - 3.6
Heneicosanoic acid	Heneicosylic acid	C21:0	<0.1 - 0.2	<0.1 - <0.1
Docosanoic acid	Behenic acid	C22:0	<0.1 - 0.5	<0.1 - 0.3
cis-13-Docosenoic acid	Erucic acid	C22:1n9	<0.1 - 1.1	<0.1 - 0.7
cis-13,16-Docosadienoic acid		C22:2	<0.1 - 2.6	<0.1 - 1.8
all-cis-4,7,10,13,16-Docosapentaenoic acid	Osbond acid	C22:5n3	<0.1 - 0.8	<0.1 - 0.7
all cis-4,7,10,13,16,19-Docosahexenoic acid	DHA	C22:6n3	0.9 - 253.9	3.3 - 149.6
Tricosanoic acid	Tricosylic acid	C23:0	<0.1 - 0.5	<0.1 - 1.2
Tetracosanoic acid	Lignoceric acid	C24:0	<0.1 - 0.2	<0.1 - 1.4
cis-15-tetracosenoic acid	Nervonic acid	C24:1n9	<0.1 - 0.4	<0.1 - 0.7

This is a provisional file, not the final typeset article

1.2 Supplementary Figures

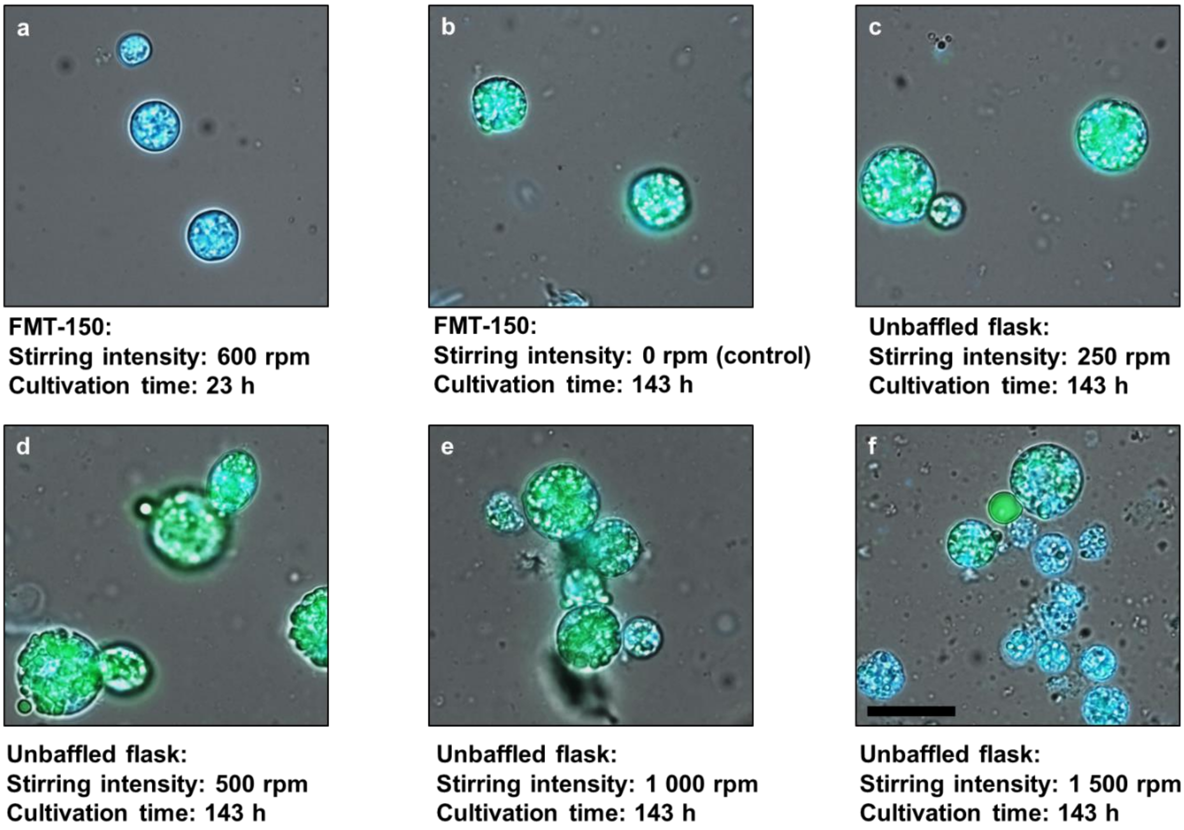

Supplementary Figure 1 The effect of shear stress on cell integrity of *Schizochytrium limacinum* CO3H. The strain was cultivated in the photobioreactor FMT-150 in a batch regime for 143 hours. Sampling was performed directly from FMT-150 at 23 h (**a**) and 143 h (**b**), and from Erlenmeyer flasks where the culture was transferred from FMT-150 at 143 h for testing the effect of shear stress (**c-f**). FMT-150 was stirred during initial 24 h at 600 rpm, for next 12 h at 300 rpm, and for the rest of the cultivation stirring was turned off. At 143 h, culture was transferred from PBR to Erlenmeyer flasks, and stirring of intensity 250 rpm (**c**), 500 rpm (**d**), 1 000 pm (**e**) and 1 500 rpm (**f**) was introduced for 60 min. Cultures were samples afterwards. After samplings, the cells were fixed by glutaraldehyde (final concentration 0.25 %) and stained by DAPI and BODIPY to visualize DNA and lipids fluorescence, respectively. The cells were photographed by Zeiss Axio Imager.M2 fluorescence microscope. DAPI fluorescence representing DNA and cell nuclei (turquoise shade of the cells on panels **a-f**) was visualized with 365 nm excitation and 445/50 nm emission filter sets. BODIPY fluorescence representing lipids (green shade of the cells) was visualized with 480 nm excitation and 535/30 nm emission filter sets. The grey objects in panels **(a-f)** represent cellular debris. With increasing stirring intensity, lipid droplets were teared out of the cells and formed lipid inclusions (green objects), that floated freely in the cultivation medium. The scale bar represents 20 µm.

This is a provisional file, not the final typeset article

2 References

Abad S, Turon X. Biotechnological production of docosahexaenoic acid using aurantiochytrium limacinum: Carbon sources comparison and growth characterization. Mar Drugs (2015) 13:7275–7284. doi: 10.3390/md13127064

Abdel-Wahab MA, El-Samawaty AERMA, Elgorban AM, Bahkali AH. Utilization of low-cost substrates for the production of high biomass, lipid and docosahexaenoic acid (DHA) using local native strain Aurantiochytrium sp. YB-05. J King Saud Univ Sci (2022) 34: doi: 10.1016/j.jksus.2022.102224

Aini UN, Lunprom S, Reungsang A, Salakkam A. Docosahexaenoic acid (DHA) production by Aurantiochytrium limacinum using cassava pulp hydrolysate as an alternative low-cost carbon source. Front Mar Sci (2022) 9:1–14. doi: 10.3389/fmars.2022.985119

Chang G, Gao N, Tian G, Wu Q, Chang M, Wang X. Improvement of docosahexaenoic acid production on glycerol by Schizochytrium sp. S31 with constantly high oxygen transfer coefficient. Bioresour Technol (2013) 142:400–406. doi: 10.1016/j.biortech.2013.04.107

Chauhan AS, Patel AK, Chen CW, Chang JS, Michaud P, Dong C Di, Singhania RR. Enhanced production of high-value polyunsaturated fatty acids (PUFAs) from potential thraustochytrid Aurantiochytrium sp. Bioresour Technol (2023) 370: doi: 10.1016/j.biortech.2022.128536

Chi Z, Liu Y, Frear C, Chen S. Study of a two-stage growth of DHA-producing marine algae Schizochytrium limacinum SR21 with shifting dissolved oxygen level. Appl Microbiol Biotechnol (2009) 81:1141–1148. doi: 10.1007/s00253-008-1740-7

Ethier S, Woisard K, Vaughan D, Wen Z. Continuous culture of the microalgae Schizochytrium limacinum on biodiesel-derived crude glycerol for producing docosahexaenoic acid. Bioresour Technol (2011) 102:88–93. doi: 10.1016/j.biortech.2010.05.021

Fan KW, Chen F, Jones EBG, Vrijmoed LLP. Eicosapentaenoic and docosahexaenoic acids production by and okara-utilizing potential of thraustochytrids. J Ind Microbiol Biotechnol (2001) 27:199–202. doi: 10.1038/sj.jim.7000169

Ganuza E, Anderson AJ, Ratledge C. High-cell-density cultivation of Schizochytrium sp. in an ammonium/pH-auxostat fed-batch system. Biotechnol Lett (2008) 30:1559–1564. doi: 10.1007/s10529-008-9723-4

Hu X, Tang X, Bi Z, Zhao Q, Ren L. Adaptive evolution of microalgae Schizochytrium sp. under high temperature for efficient production of docosahexaeonic acid. Algal Res (2021) 54:102212. doi: 10.1016/j.algal.2021.102212

Huang TY, Lu WC, Chu IM. A fermentation strategy for producing docosahexaenoic acid in Aurantiochytrium limacinum SR21 and increasing C22:6 proportions in total fatty acid. Bioresour Technol (2012) 123:8–14. doi: 10.1016/j.biortech.2012.07.068

Li J, Liu R, Chang G, Li X, Chang M, Liu Y, Jin Q, Wang X. A strategy for the highly efficient production of docosahexaenoic acid by Aurantiochytrium limacinum SR21 using glucose and glycerol as the mixed carbon sources. Bioresour Technol (2015) 177:51–57. doi: 10.1016/j.biortech.2014.11.046

This is a provisional file, not the final typeset article

Menegol T, Romero-Villegas GI, López-Rodríguez M, Navarro-López E, López-Rosales L, Chisti Y, Cerón-García MC, Molina-Grima E. Mixotrophic production of polyunsaturated fatty acids and carotenoids by the microalga Nannochloropsis gaditana. J Appl Phycol (2019) 31:2823–2832. doi: 10.1007/s10811-019-01828-3

Nazir Y, Halim H, Al-Shorgani NKN, Manikan V, Hamid AA, Song Y. Efficient conversion of extracts from low-cost, rejected fruits for high-valued Docosahexaenoic acid production by Aurantiochytrium sp. SW1. Algal Res (2020) 50: doi: 10.1016/j.algal.2020.101977

Patel A, Rova U, Christakopoulos P, Matsakas L. Assessment of fatty acids profile and omega-3 polyunsaturated fatty acid production by the oleaginous marine thraustochytrid aurantiochytrium sp. T66 cultivated on volatile fatty acids. Biomolecules (2020) 10: doi: 10.3390/biom10050694

Perveen Z, Ando H, Ueno A, Ito Y, Yamamoto Y, Yamada Y, Takagi T, Kaneko T, Kogame K, Okuyama H. Isolation and characterization of a novel thraustochytrid-like microorganism that efficiently produces docosahexaenoic acid. Biotechnol Lett (2006) 28:197–202. doi: 10.1007/s10529-005-5335-4

Qu L, Ji XJ, Ren LJ, Nie ZK, Feng Y, Wu WJ, Ouyang PK, Huang H. Enhancement of docosahexaenoic acid production by Schizochytrium sp. using a two-stage oxygen supply control strategy based on oxygen transfer coefficient. Lett Appl Microbiol (2011) 52:22–27. doi: 10.1111/j.1472-765X.2010.02960.x

Ren LJ, Ji XJ, Huang H, Qu L, Feng Y, Tong QQ, Ouyang PK. Development of a stepwise aeration control strategy for efficient docosahexaenoic acid production by Schizochytrium sp. Appl Microbiol Biotechnol (2010) 87:1649–1656. doi: 10.1007/s00253-010-2639-7

Ren LJ, Sun LN, Zhuang XY, Qu L, Ji XJ, Huang H. Regulation of docosahexaenoic acid production by Schizochytrium sp.: Effect of nitrogen addition. Bioprocess Biosyst Eng (2014) 37:865–872. doi: 10.1007/s00449-013-1057-5

Russo GL, Langellotti AL, Blasco T, Oliviero M, Sacchi R, Masi P. Production of omega-3 oil by aurantiochytrium mangrovei using spent osmotic solution from candied fruit industry as sole organic carbon source. Processes (2021) 9: doi: 10.3390/pr9101834

Sahin D, Tas E, Altindag UH. Enhancement of docosahexaenoic acid (DHA) production from Schizochytrium sp. S31 using different growth medium conditions. AMB Express (2018) 8: doi: 10.1186/s13568-018-0540-4

Shafiq M, Zeb L, Cui G, Jawad M, Chi Z. High-Density pH-Auxostat Fed-Batch Culture of Schizochytrium limacinum SR21 with Acetic Acid as a Carbon Source. Appl Biochem Biotechnol (2020) 192:1163–1175. doi: 10.1007/s12010-020-03396-6

Thurn AL, Stock A, Gerwald S, Weuster-Botz D. Simultaneous photoautotrophic production of DHA and EPA by Tisochrysis lutea and Microchloropsis salina in co-culture. Bioresour Bioprocess (2022) 9: doi: 10.1186/s40643-022-00612-5

Wang SK, Wang X, Tian YT, Cui YH. Nutrient recovery from tofu whey wastewater for the economical production of docosahexaenoic acid by Schizochytrium sp. S31. Science of the Total Environment (2020) 710:136448. doi: 10.1016/j.scitotenv.2019.136448

This is a provisional file, not the final typeset article
